# Comprehensive Analysis of m5C Methylation Regulatory Genes and Tumor Microenvironment in Prostate Cancer

**DOI:** 10.3389/fimmu.2022.914577

**Published:** 2022-06-10

**Authors:** Guopeng Yu, Jiahao Bao, Ming Zhan, Jiangyi Wang, Xinjuan Li, Xin Gu, Shangqing Song, Qing Yang, Yushan Liu, Zhong Wang, Bin Xu

**Affiliations:** ^1^Department of Urology, Shanghai Ninth People’s Hospital, Shanghai Jiaotong University School of Medicine, Shanghai, China; ^2^Hospital of Stomatology, Guanghua School of Stomatology, Sun Yat-sen University, Guangdong Provincial Key Laboratory of Stomatology, Guangzhou, China; ^3^General Medical Department, Yangpu Daqiao Community Health Service Center, Shanghai, China

**Keywords:** prostate cancer, m5C methylation, prognostic model, molecular subtype, tumor microenvironment

## Abstract

**Background:**

5-Methylcytidine (m5C) methylation is an emerging epigenetic modification in recent years, which is associated with the development and progression of various cancers. However, the prognostic value of m5C regulatory genes and the correlation between m5C methylation and the tumor microenvironment (TME) in prostate cancer remain unknown.

**Methods:**

In the current study, the genetic and transcriptional alterations and prognostic value of m5C regulatory genes were investigated in The Cancer Genome Atlas and Gene Expression Omnibus datasets. Then, an m5C prognostic model was established by LASSO Cox regression analysis. Gene set variation analyses (GSVA), gene set enrichment analysis (GSEA), clinical relevance, and TME analyses were conducted to explain the biological functions and quantify the TME scores between high-risk and low-risk subgroups. m5C regulatory gene clusters and m5C immune subtypes were identified using consensus unsupervised clustering analysis. The Cell-type Identification By Estimating Relative Subsets of RNA Transcripts algorithm was used to calculate the contents of immune cells.

**Results:**

*TET3* was upregulated at transcriptional levels in PCa compared with normal tissues, and a high *TET3* expression was associated with poor prognosis. An m5C prognostic model consisting of 3 genes (*NSUN2*, *TET3*, and *YBX1*) was developed and a nomogram was constructed for improving the clinical applicability of the model. Functional analysis revealed the enrichment of pathways and the biological processes associated with RNA regulation and immune function. Significant differences were also found in the expression levels of m5C regulatory genes, TME scores, and immune cell infiltration levels between different risk subgroups. We identified two distinct m5C gene clusters and found their correlation with patient prognosis and immune cell infiltration characteristics. Naive B cells, CD8+ T cells, M1 macrophages and M2 macrophages were obtained and 2 m5C immune subtypes were identified. *CTLA4*, *NSUN6*, *TET1*, and *TET3* were differentially expressed between immune subtypes. The expression of *CTLA4* was found to be correlated with the degree of immune cell infiltration.

**Conclusions:**

Our comprehensive analysis of m5C regulatory genes in PCa demonstrated their potential roles in the prognosis, clinical features, and TME. These findings may improve our understanding of m5C regulatory genes in the tumor biology of PCa.

## Introduction

Prostate cancer (PCa), namely prostate adenocarcinoma (PRAD), is one of major diseases that affect men’s health. It is the second most common type of cancer in men and the fifth leading cause of male cancer-related death, with an estimate of almost 1.4 million new cases and 375,000 deaths in 2020 worldwide (1, 2). Active surveillance, surgery, radiotherapy, chemotherapy, hormonal therapy, or a combination of these options are conventional methods to treat PCa patients (3, 4). The survival outcome of PCa is highly dependent on the tumor stage at diagnosis (5). Patients with localized or regional PCa often have a high 5-year survival rate, which is approximately 98% and 83% in the United States and Europe, respectively (1). However, approximately 20%–30% patients with localized PCa after treatment experience recurrence, associated with poor outcomes (6). Once metastatic PCa is detected, the 5-year survival rate is only 30%. Immune-based treatment has been a current research hotspot in PCa treatment. Immunotherapy for PCa, such as programmed death-1 (PD-1), programmed death ligand-1 (PD-L1), and cytotoxic T lymphocyte–associated antigen 4 (CTLA4) inhibitors, has achieved good results in antitumor tumor effects and become an active field of investigation in the recent 5 years (7, 8). Nevertheless, some clinical trials of immunotherapy in PCa patients have only shown modest clinical outcomes (9).

The tumor microenvironment (TME), an important part of the tumor mass, which consists of tumor cells, immune cells, and stromal cells, has been reported to promote tumor prognosis and cause drug resistance in PCa (10, 11). An increasing number of studies have observed that tumor-infiltrating immune cells in TME affect the patients’ prognosis and efficacy of immunotherapy and chemotherapy (12, 13). However, the immune mechanisms of TME in PCa remain unclear. Therefore, an investigation of the immunophenotypes and a comprehensive understanding of the TME features are urgently needed for immunotherapy improvement.

RNA methylation, a post-transcriptional modification, may impact gene expression through RNA metabolism, splicing, stability, and translation (14, 15). It has been reported that RNA methylation plays an important role in regulating a variety of biological functions and is correlated with tumor development and malignant progression (16). 5-Methylcytosine (m5C) methylation is a modification form of the fifth N of cytosine and is widely distributed in various RNAs including mRNA, tRNA, rRNA, viral RNA, vault RNA, and lncRNA, which participate in RNA stability and translation efficiency (15, 17). The formation process of m5C methylation is catalyzed by methyltransferases, also termed as “Writer” such as NSUN and DNMT family members, and can be dynamically regulated by demethylases such as TET families, and binding proteins such as YBX1, which are termed as “Eraser” and “Reader,” respectively (18). Increasing evidence demonstrated that m5C regulators play a significant role in multiple biological and pathological processes including cellular proliferation and differentiation, stem cell fate determination, embryonic development, tumorigenesis, tumor malignant progression, and tumor immunity (16, 19). Recently, studies revealed that m5C methylation regulatory genes such as YBX1 are associated with the pathogenesis of bladder urothelial carcinoma and the prognosis in patients with lung cancer or pancreatic cancer (20, 21). The alteration of m5C regulatory genes can also affect immune cells and thus reshape the TME. For instance, the TET family can impact the function of many immune cell phenotypes including B cells, plasma cells, dendritic cells, and Tregs (22–24). However, ambiguity remains about the potential functions and mechanisms of m5C methylation regulatory genes in the development of cancer, especially in PCa. In addition, the relationship between m5C methylation, TME, and tumor immunotherapy is not fully understood. Therefore, a comprehensive analysis of TME features mediated by m5C regulatory genes will further our understanding on the TME and provide important insights for immunotherapy in PCa.

In the current study, we comprehensively evaluated the genetic and transcriptional alterations of m5C regulatory genes based on the TCGA and GEO database. Then, we constructed a prognostic model and performed functional analysis. m5C regulatory gene clusters and m5C immune subtypes were identified, and the relationship between m5C regulatory genes and tumor immunity was investigated.

## Methods and Materials

### The Workflow of Study Strategies

In our study, we explored the m5C regulatory gene expression profiles between PCa and normal samples based on the TCGA database and GEO database. Then, the univariable Cox regression and LASSO Cox regression analyses were applied to identify prognostic biomarkers and develop a prognostic model. Functional enrichment analysis, clinical characteristics analysis, and immune infiltration analysis were conducted. Furthermore, we identified m5C regulatory gene clusters and m5C immune subtypes using a consensus-unsupervised clustering analysis and investigated the correlation between the subtypes and tumor immunity.

### Data Sources and Preprocessing

The gene expression data and clinical information of PCa patients were obtained from The Cancer Genome Atlas (TCGA) database (https://portal.gdc.cancer.gov/) and Gene Expression Omnibus database (https://www.ncbi.nlm.nih.gov/geo/) (**Supplementary Table 1**) (25, 26). A total of 499 PCa cases and 52 normal cases with the gene expression profile (HTSeq-FPKM), copy number variation (CNV), single-nucleotide polymorphism (SNP), and relevant clinicopathological information of prostate adenocarcinoma projects were collected from the TCGA database. Level 3 HTSeq-FPKM data were transformed into transcripts per million (TPM) reads for the subsequent analyses. Three GEO cohorts including GSE3325, GSE55945, and GSE155056 datasets were acquired from the GEO database (27–29). The gene expression data of GSE3325 (tumor=13, normal=6) and GSE55945 (tumor=11, normal=8) were based on the platform of GPL570 (Affymetrix Human Genome U13s3 Plus 2.0 Array) and GSE155056 (tumor=3, normal=3) was based on the platform of GPL28784 (085499_SBC human ceRNA microarray version 1.1). The samples in 3 GEO cohorts were all collected from human prostate benign and malignant tissues.

### Identification of Differentially Expressed m5C Regulatory Genes

m5C regulatory genes (*TET1*, *TET3*, *DNMT3B*, *YBX1*, *NSUN2*, *NSUN6*, *NOP2*) were extracted from the prior studies (18, 30). First, three GEO datasets were integrated and the “Combat” algorithm from “SVA” package of R software was employed to eliminate the batch effects caused by non-biotechnological bias (31). Then, we used the “Limma” package to identify differentially expressed genes (DEGs) between the tumor and the normal samples. |Log2(Foldchange)| >1 and an adjusted *P*-value <0.05 was regarded as the threshold to indicate DEGs. Based on all m5C regulatory gene expression levels, principal component analysis (PCA) was performed using the “prcomp” function. Results were visualized using the R package “ggplot2”.

### CNV and SNP Analysis

The Genomic Identification of Significant Targets in Cancer (GISTIC) algorithm is widely applied to identify both broad and focal (potentially overlapping) recurring events, which are associated with trigger cancer pathogenesis (32). GISTIC 2.0 software was employed to detect genes showing significant deletion and amplification in thousands of samples. An amplification or deletion length >0.1 and *P*<0.05 were considered as the parameter threshold. We utilized MutSig2.0 software to analyze the Mutation Annotation Format (MAF) file of TCGA mutation data to identify significantly mutated genes. A *P*-value <0.05 was considered significant.

### Construction of the m5C Prognostic Model in PCa

We applied m5C regulatory genes to construct a prognostic risk score signature. Firstly, the univariate Cox regression analysis was conducted to extract m5C regulatory genes that were associated with the overall survival significantly. The result was visualized by the R package “forestplot”. Then, the least absolute shrinkage and selection operator (LASSO) Cox regression analysis was performed to reduce the dimension of high-latitude data and construct the prognostic model using the R package “glmnet” (33, 34). Ten-fold cross-validation was employed to avoid the overfitting problem and select the penalty parameter (λ) according to the minimum criteria. The prognostic scoring system for PCa patients was developed based on a linear combination of regression coefficients derived from the LASSO Cox regression analysis coefficients multiplied by the expression levels of genes, and then patients were divided into high-risk and low-risk subgroups accordingly. We compared the overall survival probability of high-risk and low-risk subgroups using the R package “survival” and “jskm”. A landmark time of 6 years was set. To evaluate the stability of the prognostic model, the R package “survivalROC” was employed to perform receiver operating characteristic (ROC) analysis and calculate the value of the area under the curve (AUC). The expression levels of m5C regulatory genes were analyzed between different risk subtypes classified by the prognostic model.

### GSVA and GSEA

Gene set variation analysis (GSVA) is a non-parametric, unsupervised method for estimating the variation of gene set enrichments in the gene expression data, which is commonly used for exploring the variation in the pathway and biological process activity in the samples of an expression dataset (35). We performed GSVA to investigate the difference of the biological function between high- and low-risk subgroups. “c2.kegg.v7.4.symbols” and “c5.go.v7.4.symbols” gene sets were applied to performed GSVA using the R package “GSVA”. The R package “pheatmap” was applied to visualize the results.

Gene set enrichment analysis (GSEA) was conducted by the R package “clusterProfiler” to determine whether prior-defined functional or pathway sets of genes differ significantly between high- and low-risk subgroups in the enrichment of MSigDB Collection (“c2.kegg.v7.4.symbols” and “c5.go.v7.4.symbols” gene sets) (http://www.gsea-msigdb.org/gsea/index.jsp) (36). The enrichments of gene sets with a *P*-value <0.05 were regarded to be significant.

### Correlation of m5C Prognostic Model with TME and Clinical Features

The R package “ESTIMATE” can calculate the TME scores including the stromal score, immune score, and estimate score using gene expression datasets and evaluate the relative contents of stromal cells or immunocytes in the TME. Higher stromal scores or immune scores suggest higher relative contents of stromal cells or immunocytes. Estimate scores represent the aggregation of stromal scores and immune scores (37). We investigate the correlation between TME scores and high- or low-risk subgroups.

To individualize the predicted PCa patients’ survival probability for 1, 3, and 5 years, a nomogram was developed using the R package “regplot.” The nomogram contained the risk score of the m5C prognostic model and clinical characteristics including the age, M stage, and T stage. Then, we conducted calibration analysis and calculated the optimism-corrected concordance index (C-Index) by 1,000 bootstrap resamples to assess the discrimination of the predictive nomogram.

### Identification of m5C Regulatory Gene Clusters in PCa

Based on the expression of m5C regulatory genes, we performed consensus unsupervised clustering analysis to classify PCa patients into distinct gene clusters employing R package “ConsensusClusterPlus”, with the parameters of reps = 1,000 and pItem = 0.8 (38). To verify the stability of classification, PCA was conducted based on the expression of all m5C regulatory genes and the R package “ggplot2” was used to visualize the results. Furthermore, we investigated the relationship of the m5C prognostic model, m5C regulatory gene clusters, and clinical pathological features.

### Estimation of TME Cell Infiltration

Cell-type Identification by Estimating Relative Subsets of RNA Transcripts (CIBERSORT) is a de-convolution algorithm that uses a set of reference gene expression matrixes to evaluate 22 immune cell-type proportions from bulk tumor sample expression data based on support vector regression. We conducted CIBERSORT analysis using the R package “CIBERSORT” to investigate TME cell infiltration between different groups. The relative contents of immune cells were calculated in distinct risk score subtypes and m5C regulatory gene clusters by the CIBERSORT algorithm.

### Identification of m5C Immune Subtypes and Cancer Immunotherapy Analysis

Based on the relative contents of immune cells, which were significantly different between the high- and low-risk subgroups classified by the m5C prognostic model, as well as between different m5C regulatory gene clusters, we performed consensus unsupervised clustering analysis to classify PCa patients into distinct immune subtypes employing the R package “ConsensusClusterPlus”, with the parameters of reps = 50 and pItem = 0.8 (38). Meanwhile, we explore the expression levels of m5C regulatory genes between the different m5C immune subtypes.

The cytotoxic T lymphocyte–associated protein 4 (CTLA4) gene has been demonstrated to be a key immunotherapy related gene (39). To determine whether CTLA4 plays a role in immunotherapy for PCa patients through TME cell infiltration, we investigated the correlation of the expression of CTLA4 and m5C immune subtypes and infiltration levels for different immune cells.

### Statistical Analysis

All statistical analyses were conducted with R software (v4.0.2). To compare the variables between the 2 groups, we employed the independent sample t tests for normally distributed continuous variables and the Wilcoxon rank sum test (Mann–Whitney U tests) for nonnormally distributed continuous variables. All tests were 2 sided, and *P* < 0.05 was considered statistically significant.

## Results

### Genetic and Transcriptional Alterations of m5C Regulatory Genes in PCa

**Figure 1:**
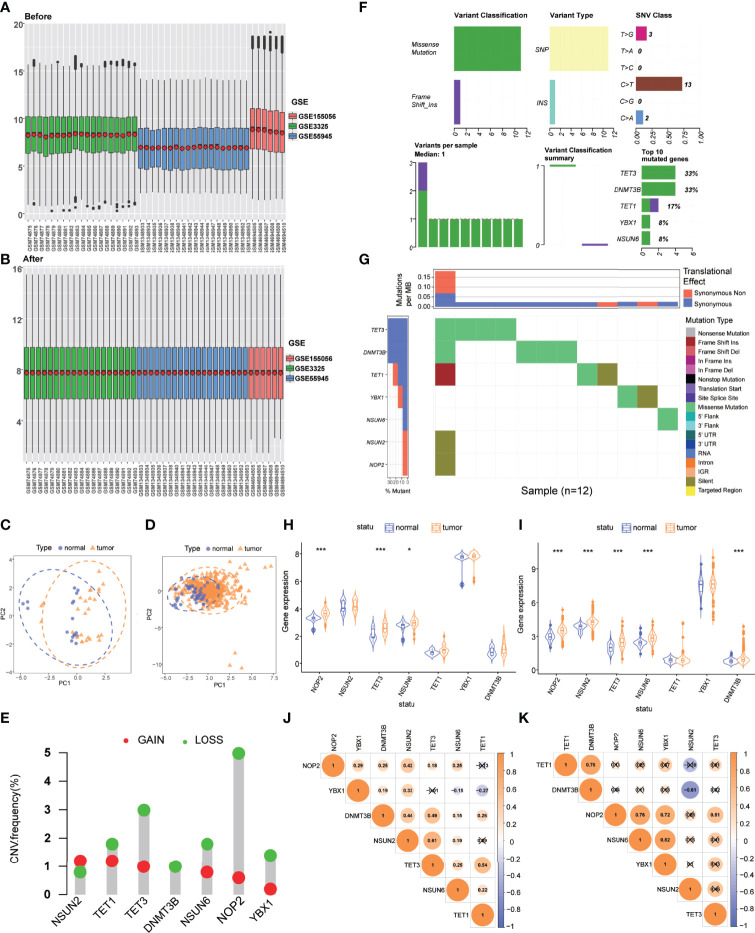
Genetic and transcriptional alterations of m5C regulatory genes in PCa. **(A)** Gene expression levels of three GEO datasets before integration. **(B)** Gene expression levels of three GEO datasets after integration. **(C)** PCA for the expression profiles of m5C regulatory genes to distinguish tumors from normal samples in the integrated GEO dataset (normal, blue; tumor, orange). **(D)** PCA for the expression profiles of m5C regulatory genes to distinguish tumors from normal samples in the TCGA dataset (normal, blue; tumor, orange). **(E)** CNV amplifications and deletions of m5C regulatory genes. **(F)** The landscape of genetic variation of m5C regulatory genes. **(G)** SNV categories and frequencies of m5C regulatory genes. **(H)** The expression of 7 m5C regulatory genes between tumor and normal samples in the integrated GEO dataset. **(I)** The expression of 7 m5C regulatory genes between tumor and normal samples in the TCGA dataset. **(J)** Co-expression analysis of m5C regulatory genes in the integrated GEO dataset. **(K)** Co-expression analysis of m5C regulatory genes in the TCGA dataset. *P*-values were shown as *P < 0.05 and ***P < 0.001.

Three GEO datasets were enrolled into one integrated dataset, and the batch effects were eliminated (**Figures 1A, B**). **Figures 1C, D** showed the result of PCA conducted in the integrated GEO dataset and TCGA dataset, respectively, which revealed significant differences in the m5C regulatory genes transcription profiles between PCa and normal samples. Seven m5C regulatory genes (*TET1*, *TET3*, *DNMT3B*, *YBX1*, *NSUN2*, *NSUN6*, *NOP2*) were extracted for subsequent analyses. Differential expression analysis, SNP analysis, and co-expression analysis were performed. CNV frequencies were computed based on the data from TCGA to identify genes with significant amplifications or deletions. Among m5C regulatory genes, *NOP2* exhibited the highest amplification frequency and lowest deletion frequency (**Figure 1E**). **Figure 1F** exhibited the landscape of SNP and mutations in PCa samples. **Figure 1G** showed the m5C regulatory gene alterations closely associated with PCa progression, including frameshift insertion, frameshift deletion, missense mutation, synonymous mutation, nonsense mutation, and other non-synonymous or cleavage sites. *NOP2*, *TET3*, *NSUN6*, and *DNMT3B* were identified as differentially expressed genes (DEGs) (*P* < 0.05) (**Figures 1H, I**). Co-expression analysis showed that varying degrees of correlation existed among m5C regulatory genes (**Figures 1J, K**).

### Construction of the m5C Prognostic Model in PCa

**Figure 2:**
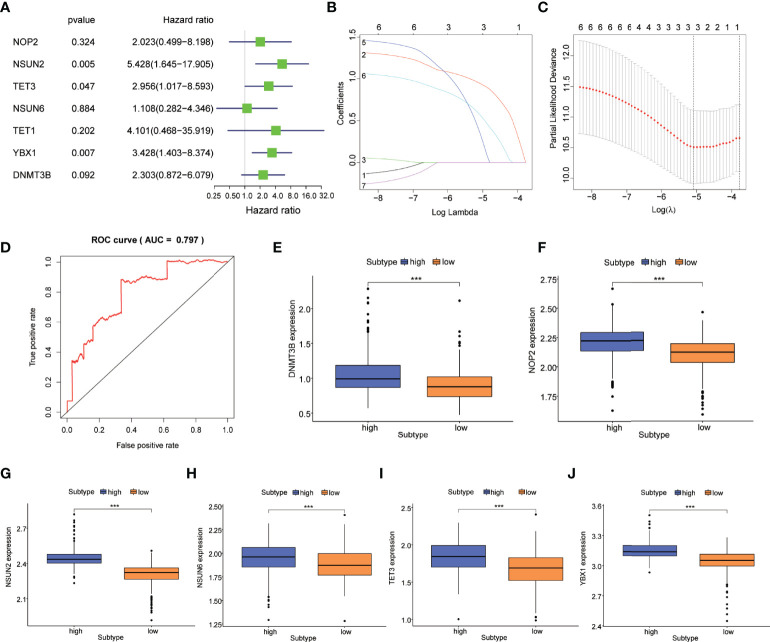
Construction of the m5C prognostic model in PCa. **(A)** m5C regulatory genes related to the prognosis of PCa were identified by univariable Cox regression in a forest plot. **(B)** LASSO Cox regression analysis of m5C regulatory genes. **(C)** The parameter selection was tuned by cross-validation in the LASSO Cox regression. **(D)** ROC curves showed the prognostic performance of the m5C prognostic model. Difference in the expression of m5C regulatory genes including **(E)** *DNMT3B*, **(F)** *NOP2*, **(G)** *NSUN2*, **(H)** *NSUN6*, **(I)** *TET3*, and **(J)** *YBX1* between high-risk and low-risk subtypes. *P*-values was shown as ***P < 0.001.

We conducted univariate Cox regression analysis to investigate the prognostic value of m5C regulatory genes and screened *NSUN2* (HR=5.428, 95%CI=1.645-17.905, *P*=0.005), *TET3* (HR=2.956, 95%CI=1.017-8.593, *P*=0.047), and *YBX1* (HR=3.428, 95%CI=1.403-8.347, *P*=0.007) that were associated with the prognosis of PCa (**Figure 2A**; **Supplementary Table 2**).

M5C regulatory genes were fit in the LASSO Cox regression analysis, and three genes including *NSUN2*, *TET3*, and *YBX1* were selected to develop an m5C prognostic model based on the optimal value of λ (**Figures 2B, C**). PCa patients were classified into high-risk and low-risk subtypes according to the risk scores calculated by the model. **Supplementary Figure 1A** showed the distribution of the risk score and cut-off value in the TCGA cohort. As the survival curves crossed, we used landmark survival analysis to compare the difference between the different risk subtypes (**Supplementary Figure 1B**). The result of landmark analysis survival showed a longer OS in PCa patients in the low-risk subtype within 6 years (*P* = 0.014). Nevertheless, no significant difference was found in the survival probability beyond 6 years (*P* = 0.578). Subsequently, the ROC curve was plotted to assess the accuracy of our model’s predictions. As shown in **Figure 2D**, the AUC of the model was 0.797, which suggested a good efficacy in prognostic prediction. The expression levels of m5C regulatory genes between high-risk and low-risk subtypes were investigated. *DNMT3B*, *NOP2*, *NSUN2*, *NSUN6*, *TET3*, and *YBX1* were differentially expressed (*P<*0.001) (**Figures 2E–J**).

**Figure 3:**
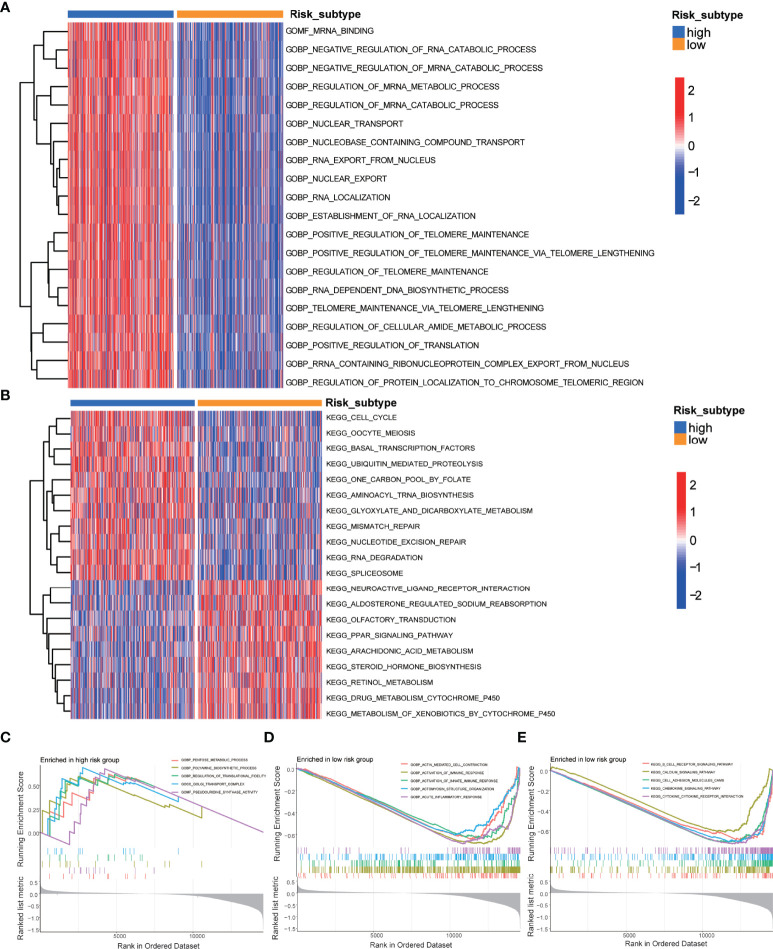
GSVA and GSEA of the m5C prognostic model. **(A)** GSVA of GO terms between high-risk and low-risk subtypes, in which red and blue represent activated and inhibited biological processes, respectively. **(B)** GSVA of KEGG terms between high-risk and low-risk subtypes, in which red and blue represent activated and inhibited pathways, respectively. **(C)** GSEA of the significantly enriched GO terms in the high-risk subtype. **(D)** GSEA of the significantly enriched GO terms in the low-risk subtype. **(E)** GSEA of the significantly enriched KEGG terms in the low-risk subtype.

Then, to further explore the functional annotation between high-risk and low-risk subtypes, GSVA was performed (**Supplementary Table 3**). The results of the GSVA of gene ontology biological processes (GOBPs) showed that the high-risk subtype was significantly enriched in the regulation of protein localization to the chromosome telomeric region, positive regulation of telomerase RNA localization to the cajal body, telomerase RNA localization, IMP biosynthetic process, and protein localization to nucleoplasm (**Figure 3A**). As for KEGG terms in GSVA, the high-risk subtype was enriched in the nucleobase biosynthetic process, aminoacyl tRNA biosynthesis, spliceosome, mismatch repair, glyoxylate and dicarboxylate metabolism, and RNA degradation (**Figure 3B**). Gene set enrichment analysis (GSEA) was employed to identify the biological processes and signaling pathways involved in PCa between high- and low-risk subtypes. The most significantly enriched biological processes and signaling pathways are shown in **Figures 3C–E** and **Supplementary Table 4**. The risk score based on the m5C prognostic model was enriched in GOBP terms including the G protein coupled purinergic nucleotide receptor signaling pathway, G protein–coupled purinergic nucleotide receptor activity, sialic acid binding, T-cell receptor complex, and negative regulation of interleukin 8 production and KEGG terms including the B-cell receptor signaling pathway, calcium signaling pathway, cell adhesion molecules cams, chemokine signaling pathway, and cytokine–cytokine receptor interaction.

**Figure 4:**
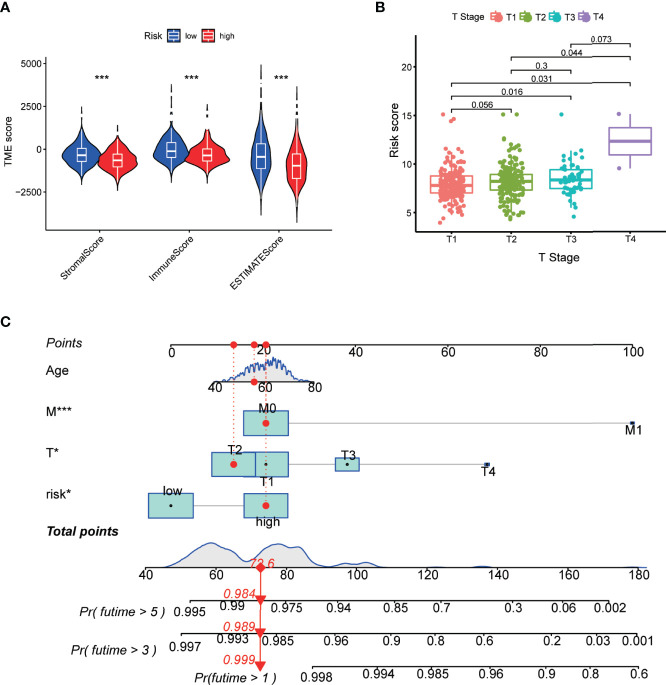
Correlation of the m5C prognostic model with TME and clinical features. **(A)** Correlation between risk score and TME scores including stromal, immune, and ESTIMATE scores. **(B)** Risk scores of the PCa patients are classified by the T stage. **(C)** Nomogram consisting of the risk score and clinical characteristics to predict the overall survival in the PCa patients. *P*-values were shown as *P < 0.05 and ***P < 0.001.

Furthermore, we evaluated the TME scores of high-risk and low-risk subtypes using the Estimation of STromal and Immune cells in MAlignant Tumor tissues using Expression data (ESTIMATE) algorithm. The results demonstrated that the patients in the high-risk subtype have a lower tumor immune infiltration level than those in the low-risk subtype (**Figure 4A**). Correlation analysis was performed to analyze the relationship between clinical features and risk scores. We observed that the risk score of PCa patients increased with the T stage (**Figure 4B**). We constructed a nomogram containing the risk score and clinical characteristics including the age, T stage, and M stage (**Figure 4C**). The risk score, T stage, and M stage were significantly associated with the prognosis of patients.

### Identification of m5C Regulatory Gene Clusters in PCa

**Figure 5:**
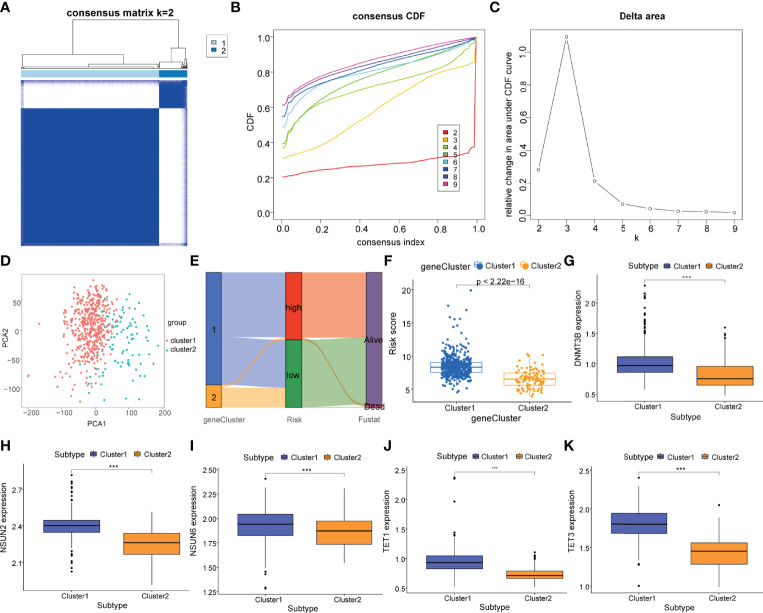
Identification of m5C regulatory gene clusters in PCa. **(A)** The PCa patients were stratified into 2 clusters based on the consensus clustering matrix (k = 2). **(B, C)** Consensus clustering model with cumulative distribution function (CDF) by k from 2 to 9. **(D)** The PCA analysis of the m5C regulatory gene cluster 1 and m5C regulatory gene cluster 2 based on the m5C regulatory genes. **(E)** Sankey diagram of subtype distributions in clusters with different risk scores and survival outcomes. **(F)** Differences in the risk score between the m5C regulatory gene cluster 1 (cluster 1, blue) and the m5C regulatory gene cluster 2 (cluster 2, yellow). Expression of m5C regulatory genes including **(G)** *DNMT3B*, **(H)** *NSUN2*, **(I)** *NSUN6*, **(J)** *TET1*, and **(K)** *TET3* in m5C regulatory gene cluster 1 (cluster 1, blue) and m5C regulatory gene cluster 2 (cluster 2, yellow). *P*-values was shown as ***P < 0.001.

To further explore the potential biological characteristics of m5C regulatory genes in PCa patients, a consensus clustering algorithm was employed to classify patients into two distinct modification patterns based on the expression of 7 m5C regulatory genes, including 458 cases in modification pattern 1 and 93 patients in the modification pattern 2, which were termed as m5C regulatory gene cluster 1 and 2 (**Figures 5A–C**). The PCA plots both demonstrated an obvious different distribution between two clusters (**Figure 5D**). Then, we used a Sankey diagram to visualize the relationship between gene clusters, risk score subtypes, and the survival status (**Figure 5E**). We observed that the patients in m5C gene cluster 1 corresponded with the high-risk subtype, which dominated most of the PCa patients with a death status. Compared to gene cluster 2, gene cluster 1 had a significantly higher risk score (*P*<2.2e-16, **Figure 5F**). In addition, the association between the expression levels of m5C regulatory genes and m5C regulatory gene clusters was investigated. **Figures 5G–K** showed that *DNMT3B*, *NSUN2*, *NSUN6*, *TET1*, and *TET3* were differentially expressed with a statistical significance between gene cluster 1 and gene cluster 2 (*P <*0.001).

### Relationship of m5C Regulatory Genes and Tumor Immune Infiltration

**Figure 6:**
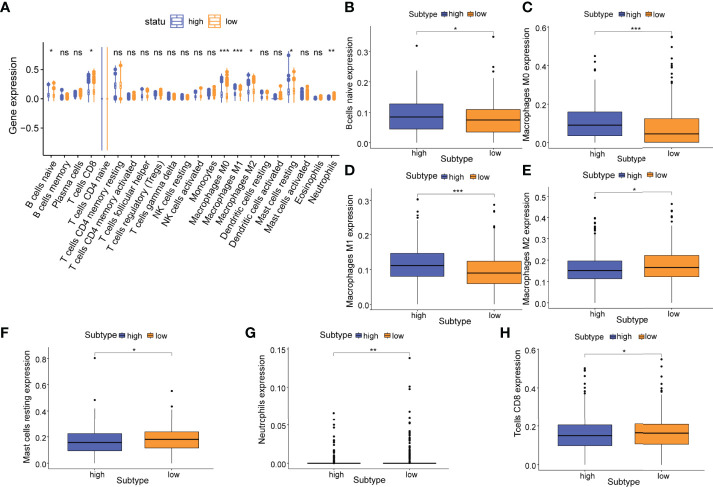
Relationship of the m5C prognostic model and tumor immune infiltration. **(A)** Differences in the abundance of 22 infiltrating immune cell phenotypes in high-risk and low-risk subtypes. Differences in the abundance of 7 infiltrating immune cell phenotypes including **(B)** naive B cells; **(C)** M0 macrophages; **(D)** M1 macrophages; **(E)** M1 macrophages; **(F)** resting mast cells; **(G)** neutrophils, and **(H)** CD8+ T cells in high-risk and low-risk subtypes. P-values were shown as follows: ns, not significant, *P < 0.05; **P < 0.01; ***P < 0.001.

To analyze the difference of tumor immune infiltration between high-risk and low-risk subtypes, we used the CIBERSORT algorithm to estimate the proportions of 22 distinct immune cell phenotypes between different subtypes. The analysis result of 22 kinds of cells showed B-cell naïve (*P*<0.05), plasma cells (*P*<0.05), macrophages M0 (*P*<0.001), macrophages M1 (*P<*0.001), and macrophages M2 (*P*<0.05), mast cells resting (*P*<0.05) and neutrophils (*P*<0.01) were statistically different between high-risk and low-risk subtypes (**Figures 6A–H**).

**Figure 7:**
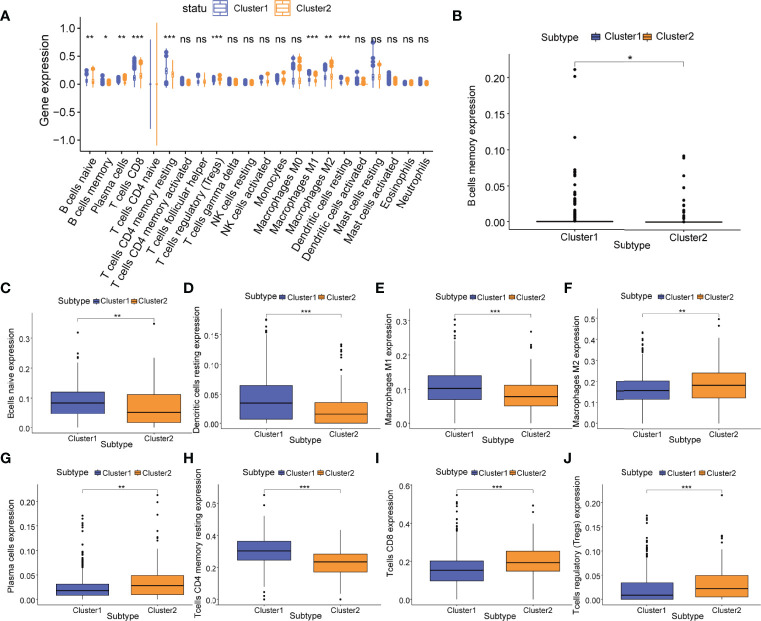
Relationship of m5C regulatory gene clusters and tumor immune infiltration. **(A)** Abundance of 22 infiltrating immune cell phenotypes in m5C regulatory gene cluster 1 (cluster 1, blue) and m5C regulatory gene cluster 2 (cluster 2, yellow). Differences in the abundance of 9 infiltrating immune cell phenotypes including **(B)** memory B cells; **(C)** naive B cells; **(D)** resting dendritic cells; **(E)** M1 macrophages; **(F)** M2 macrophages; **(G)** plasma cells; **(H)** resting CD4+ T cells; **(I)** CD8+ T cells; and **(J)** Tregs in m5C regulatory gene cluster 1 (cluster1, blue) and m5C regulatory gene cluster 2 (cluster2, yellow). P-values were shown as follows: ns, not significant, *P < 0.05; **P < 0.01; ***P < 0.001.

Moreover, between m5C regulatory gene cluster 1 and m5C regulatory gene cluster 2, we identified 9 immune cell phenotypes that were statistically different, including naive B cells (*P*<0.01), memory B cells (*P*<0.05), plasma cells (*P*<0.01), CD8 T cells (*P<*0.001), resting CD4 T cells (*P*<0.001), Tregs (*P*<0.001), M1 macrophages (*P<*0.001), M2 macrophages (*P<*0.01), and resting dendritic cells (*P*<0.001) (**Figures 7A–J**). We extracted 4 overlapping immune cell phenotypes (naive B cells, CD8 T cells, M1 macrophages, M2 macrophages) for subsequent analyses.

### Identification of m5C Immune Subtypes in PCa

**Figure 8:**
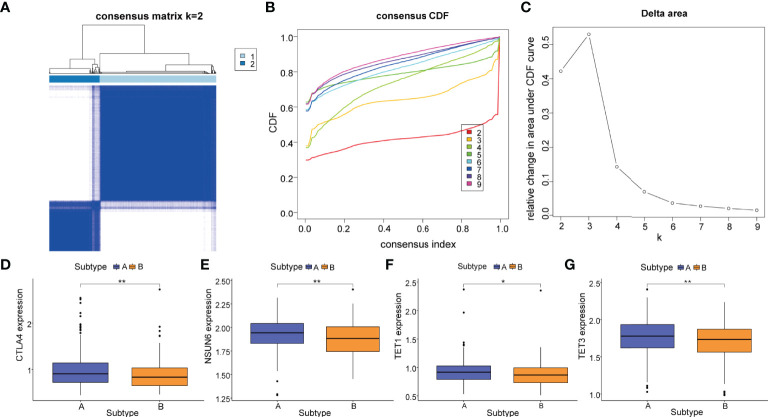
m5C immune subtypes based on key immune cell phenotypes. **(A)** The PCa patients were stratified into 2 subtypes based on the consensus clustering matrix (k = 2). **(B, C)** Consensus clustering model with cumulative distribution function (CDF) by k from 2 to 9. **(D)** The gene mRNA expressions of the immune checkpoint gene CTLA4 between m5C immune subtype A and m5C immune subtype. The gene mRNA expressions of m5C regulatory genes including **(E)** NSUN6, **(F)** TET1, and **(G)** TET3 between m5C immune subtype A (subtype A, blue) and m5C immune subtype B (subtype B, yellow). P-values were shown as follows: *P < 0.05 and **P < 0.01.

In order to further explore the correlation between key immune cell phenotypes and m5C in PCa, the R package “ConsensusClusterPlus” was used once again to classify patients based on 4 overlapping immune cell phenotypes (naive B cells, CD8 T cells, M1 macrophages, M2 macrophages) that were observed to be significantly different. PCa patients were divided into two m5C immune subtypes (subtype A, n=385; subtype B, n=166) (**Figures 8A–C**). Then, we conducted differential expression analysis for the immune checkpoint gene CTLA4 and m5C regulatory genes; we found that the expression of CTLA4 (*P*<0.01), *NSUN6* (*P*<0.01), *TET1* (*P*<0.05), and *TET3* (*P*<0.01) differed significantly between the m5C immune subtype A and B (**Figures 8D–G**).

### Correlation Between Immune Cell Phenotypes and Immune Checkpoint Gene *CTLA4*

**Figure 9:**
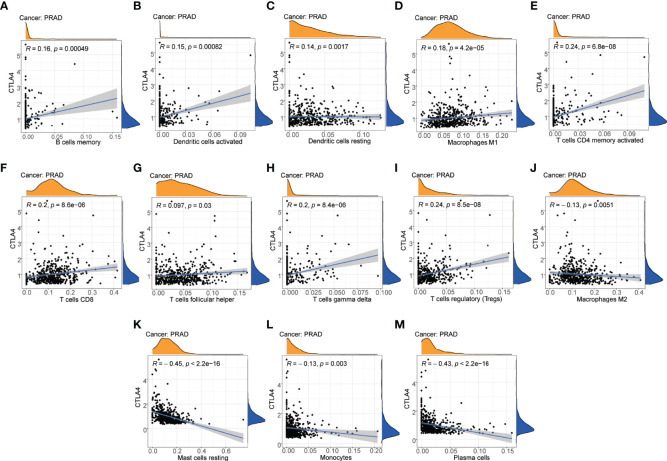
Correlation between immune cell phenotypes and immune checkpoint gene *CTLA4*. **(A)** Correlation between B cells memory and *CTLA4*. **(B)** Correlation between dendritic cells activated and *CTLA4*. **(C)** Correlation between dendritic cells resting and *CTLA4*. **(D)** Correlation between macrophages M1 and *CTLA4*. **(E)** Correlation between T-cell CD4 memory activated and *CTLA4*. **(F)** Correlation between T-cell CD8 and *CTLA4*. **(G)** Correlation between T cells follicular helper and *CTLA4*. **(H)** Correlation between T cells gamma delta and *CTLA4*. **(I)** Correlation between T cells regulatory (Tregs) and *CTLA4*. **(J)** Correlation between macrophages M2 and *CTLA4*. **(K)** Correlation between mast cells resting and *CTLA4*. **(L)** Correlation between monocytes and *CTLA4*. **(M)** Correlation between plasma cells and *CTLA4*.

To reveal a potential correlation between the infiltration of immune cell phenotypes and the efficacy of immunotherapy, we performed the CIBERSORT algorithm to assess the association between the immune checkpoint gene *CTLA4* and the abundance of immune cell phenotypes. As shown in the scatter diagrams (**Figure 9**), *CTLA4* was positively correlated with many types of innate and acquired immune cell types including memory B cells (R=0.16, *P*=000049), activated dendritic cells (R=0.15, *P*=000082), resting dendritic cells (R=0.14, *P*=000017), M1 macrophages (R=0.18, *P*=4.2e-05), CD4 T cells (R=0.24, *P*=6.8e-08), CD8 T cells (R=0.2, *P*=8.6e-06), T follicular helper cells (R=0.097, *P*=0.03), gamma T cells (R=0.2, *P*=8.4e-06), and Tregs (R=0.24, *P*=8.5e-08). CTLA4 was negatively correlated with M2 macrophages (R=-0.13, *P*=0.0051), resting mast cells (R=-0.45, *P*<2.2e-16), monocytes (R=-0.13, *P*=0.003), and plasma cells (R=-0.43, *P*<2.2e-16).

## Discussion

PCa is one of the most common malignancies in humans. Although the treatment of PCa (chemotherapies, antiandrogens, and radiopharmaceuticals) has achieved much over the past decade, the rates of tumor metastasis and recurrence remain high and were associated with a poor prognosis. It is urgent to identify new prognostic biomarkers and therapeutic strategies. m5C methylation modification is one of the most prominent modifications in eukaryotes, which contributes to epigenetic gene regulation through a different mechanism (40, 41). Increasing evidence suggested that m5C modification plays an indispensable role in both physiological and pathological processes, particularly in the development and progression of cancer (42). Many studies have explored the roles of m5C regulatory genes and constructed prognostic models in colon adenocarcinoma, hepatocellular cancer, cervical cancer, and clear cell renal cell carcinoma (43–45). However, there were a few studies that investigated the biological role of m5C regulatory genes in PCa.

In the current study, we comprehensively investigated the potential function of 7 m5C regulatory genes in PCa in muti-datasets and developed a gene signature to predict the prognosis of PCa patients. We found that *TET3* was differentially expressed between normal and tissue samples, and its high expression was associated with a poor prognosis in PCa. Compared to other genes (*TET1* and *TET2*) in the TET family, the functions of *TET3* in human cancer remains limited. It has been confirmed that *TET3* functions as a tumor promoter or tumor suppressor in different cancers and the different expression level of TET3 was associated with patients’ survival (46). A recent study found that *TET3* was also proven to overexpress in AML patients, which promoted AML growth and epigenetically regulates glucose metabolism and leukemic stem cell–associated pathways (47). The upregulation of *TET3* was proven to elevate the 5-hmc levels of the promoter regions of *c-Myc* and promoted the progression of glioma (48). These studies are in line with our results, pointing out that *TET3* has a positive association with patients’ prognosis, which could be a potential biomarker in PCa. However, the potential mechanisms still need to be explored in the further studies. Then, we developed an m5C prognostic model in PCa by the LASSO Cox regression, which contains *NSUN2*, *TET3*, and *YBX1* (AUC=0.797). It was also presented as an independent predictor for overall survival, and the risk score was increased with the T stage. Therefore, the m5C prognostic model and clinicopathological-based nomogram was established to predict the prognosis of PCa patients.

Immune-based treatment has emerged in recent years for PCa patients, which has revolutionized cancer therapy and improved patients’ overall survival in many solid tumors. However, prostate cancer, especially metastatic castration–resistant prostate cancer, was regarded as a “cold” tumor with a low immune score and massive immunosuppressive components including Tregs and TGF-β, which means that patients are more likely to suffer immunotolerance and a poor response to immunotherapy (49–51). Due to the immune-suppressive microenvironment and heterogeneous presence in PCa, it is important to identify the molecular subtypes and investigate the characteristics of the TME, which can help us predict a response to immunotherapy and recognize high-risk patients to intervene early. Previous studies showed that m5C methylation modification can affect the quantity and quality of immune cells and thus reshape the TME and impact of the efficacy of immunotherapy (52). Hence, we investigated the TME status between high-risk and low-risk subtypes. The results of GSEA demonstrated that the function of the 3 m5C regulatory genes was potentially associated with the tumor immunity biological process including the “activation of immune response”, “activation of innate immune response”, and “acute inflammatory response.” The KEGG terms of the “B cell receptor signaling pathway”, “chemokine signaling pathway”, and “cytokine cytokine receptor interaction” were also enriched in the low-risk subtype. The TME score (StromalScore, ImmuneScore, and ESTIMATEScore) in the low-risk subtype was higher than in the high-risk subtype. Meanwhile, the abundance of immune cells showed the difference between high-risk and low-risk subtypes.

To further explore the biological features of m5C regulatory genes in PCa, we revealed 2 distinct m5C gene clusters based on gene expression profiles. These 2 gene clusters exhibited different TME features. The high-risk subtype and m5C gene cluster 1 were associated with a higher infiltration of naive B cells and M1 macrophages. The low-risk subtype and m5C gene cluster 2 were associated with a higher infiltration of CD8+T cells and M2 macrophages. These differences suggested a complex biological function for infiltrating immune cells in the PCa development and progression. We identified naive B cells, CD8+ T cells, M1 macrophages, and M2 macrophages as key immune cells, and then PCa patients were classified into 2 distinct m5C immune subtypes based on the contents of key immune cells. In m5C gene cluster 1, the content of plasma cells was lower compared with m5C gene cluster 2. The difference of the proportion of plasma cells and naive B cells between two m5C gene clusters suggest that there might be an inhibitor in the TME that can block the activation of immune cells. CD8+ T-cell tumor infiltration is thought to be the key characteristics of effective immunotherapy in cancer patients, which was supposed to be associated with good response to immune checkpoint inhibitors and inhibiting the process of cancer (53, 54). However, the prognostic significance of CD8+ T cells in PCa remains controversial (55). Many studies have shown that the level of CD8+ T cell infiltration was positively correlated with cancer prognosis and responsiveness to immunotherapy (56, 57). It was reported that the CD8+ T cell subpopulation also presented immune suppressive activity in PCa. Kiniwa et al. found that CD8+ Foxp3+ regulatory T cells can mediate immunosuppression in prostate cancer (58). Tumor-associated macrophages were mainly classified into two types (M1 macrophages and M2 macrophages), which play an important role in the tumor growth and metastasis (59). In addition, the proportion of M1 macrophages and M2 macrophages can impact tumor therapy (60, 61). Previous studies showed that M1 macrophages were often known to inhibit tumor progression. Zhang et al. demonstrated that a low infiltration of M1 macrophages was associated with a poor outcome and Sadasivan’s research found that patients with high infiltration levels of M2 macrophages had an almost 5-fold increased risk of recurrence in PCa patients (62). The proportion of M1 macrophages and M2 macrophages decreased in the low-risk subtype and cluster 2, potentially shedding light on the lack of clinical achievement of immunotherapy for PCa patients (63). Targeting tumor-associated macrophages could enhance the response to other synergistical immunotherapy treatments, which provides a promising target for immunotherapy for PCa patients. These findings strongly implied the potential roles of m5C regulatory genes in reshaping the TME in PCa.

Previous trails showed a great antitumor activity for the *CTLA4* antibody ipilimumab in PCa patients (64, 65). It was demonstrated that ipilimumab not only increased the infiltration of T cells, but it can also induce immune inhibitor pathways and suppress the T-cell response (66). To explore the potential correlation between immune cells and the efficacy of the *CTLA4* inhibitor, the immune checkpoint gene *CTLA4* expression profile was analyzed. We observed higher expression levels of *CTLA4* in the m5C immune subtype A, in which the m5C regulatory genes *NSUN6*, *TET1*, and *TET3* showed a higher expression. *CTLA4* was positively correlated with memory B cells, activated dendritic cells, resting dendritic cells, M1 macrophages, CD4+ T cells, CD8+ T cells, gamma T cells, and Tregs in PCa. In addition, *CTLA4* was negatively correlated with M2 macrophages, resting mast cells, monocytes, follicular helper T cells, and plasma cells in PCa. These results suggested that *CTLA4* was implicated in the immunity of PCa. T-cell immunity and T-cell antitumor responses can be increased *via* the blockade of *CTLA4*, which is an important mechanism for immune checkpoint therapy (67).

Our study is, to our knowledge, the first to investigate the potential roles of m5C regulatory genes in cancer prognosis and tumor immunity in PCa. However, there are still some limitations in our study. The data used were obtained from public databases, and the samples used in our study were obtained retrospectively. Hence, prospective studies are needed to verify the findings in our study. Furthermore, the results of our analysis lack experiment validation and externally clinical cohort validation. The expression levels and molecular mechanisms of model genes should be further experimentally investigated.

## Conclusion

In summary, we have systematically demonstrated the expression profile, potential role, and prognostic value of m5C regulatory genes in PCa. *TET3* may serve as a potential biomarker, and a 3-gene signature was established in PCa. We identified 2 m5C gene clusters and 2 m5C immune subtypes and revealed an extensive regulatory mechanism by which m5C regulatory genes can impact the TME in PCa. These findings may improve our understanding of m5C regulatory genes in the tumor biology of PCa.

## Data Availability Statement

The analyzed data could be obtained at TGCA (https://portal.gdc.cancer.gov/) and GEO (https://www.ncbi.nlm.nih.gov/geo/) databases. The code applied in the study is available from the corresponding author on reasonable request. The accession number(s) can be found in the article/supplementary material.

## Author Contributions

All authors approved the final manuscript. BX, ZW, YL, and QY performed the study concept and design and revised the manuscript. GY, JB, MZ, and JW conducted bioinformatics analysis, performed the experiments, and wrote the manuscript. XL, XG, and SS helped to analyze data and write the manuscript.

## Funding

The study is sponsored by Natural Science Foundation of Shanghai (22ZR1437300) to GY, the Interdisciplinary Program of Shanghai Jiao Tong University (YG2019QNA12) to GY, the Shanghai Anticancer Association EYAS PROJECT (SACA-CY19C03) to GY, Fundamental Research Program Funding of Ninth People’s Hospital affiliated to Shanghai Jiao Tong University School of Medicine(JYZZ006) to GY, the National Natural Science Foundation of China (82072846, M-0171) to BX, the Innovative research team of high-level local universities in Shanghai (19DZ2204000) to BX, and the Project of Biobank (YBKB201904) from Shanghai Ninth People’s Hospital, Shanghai Jiao Tong University School to ZW.

## Conflict of Interest

The authors declare that the research was conducted in the absence of any commercial or financial relationships that could be construed as a potential conflict of interest.

The reviewer DC declared a shared parent affiliation with the author JB to the handling editor at the time of the review.

## Publisher’s Note

All claims expressed in this article are solely those of the authors and do not necessarily represent those of their affiliated organizations, or those of the publisher, the editors and the reviewers. Any product that may be evaluated in this article, or claim that may be made by its manufacturer, is not guaranteed or endorsed by the publisher.
