# Supplementary material for: Comprehensive Analysis of m5C Methylation Regulatory Genes and Tumor Microenvironment in Prostate Cancer
Source: Front Immunol. 2022 Jun 10;13:914577. doi: 10.3389/fimmu.2022.914577 (PMC9226312; doi:10.3389/fimmu.2022.914577)
Supplement: Supplementary file 1 [file DataSheet_1.docx]

Supplementary Material

# Supplementary Figures


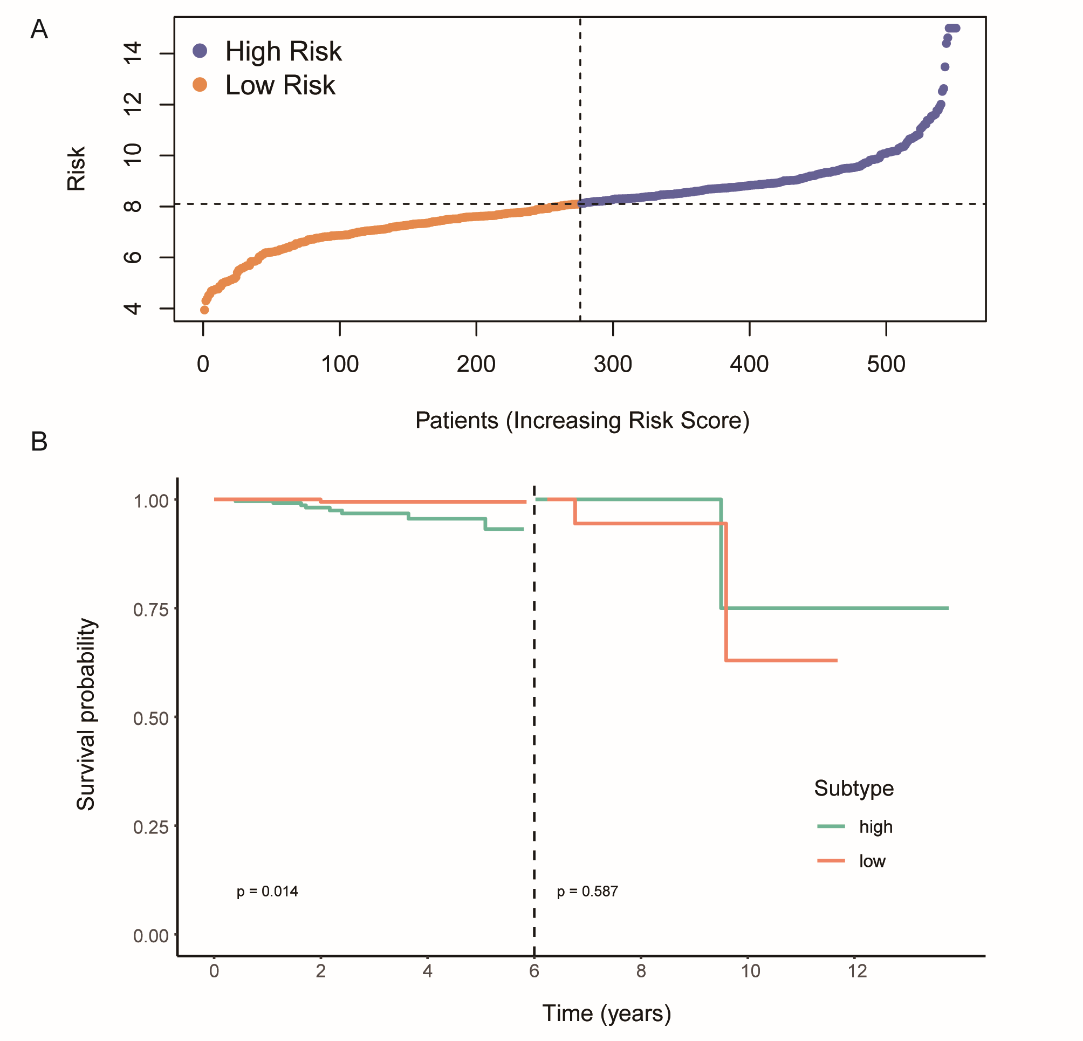


**Supplementary Figure 1.** Correlation of risk score with clinical outcomes. (A) Distribution of risk score in TCGA cohort. (B) Landmark survival analysis for different risk subtypes. The overall survival probablity of PCa patients in the high-risk and low-risk subtypes was calculated by Kaplan-Meier analysis. A landmark time of 6 years was set.

# Supplementary Tables

**
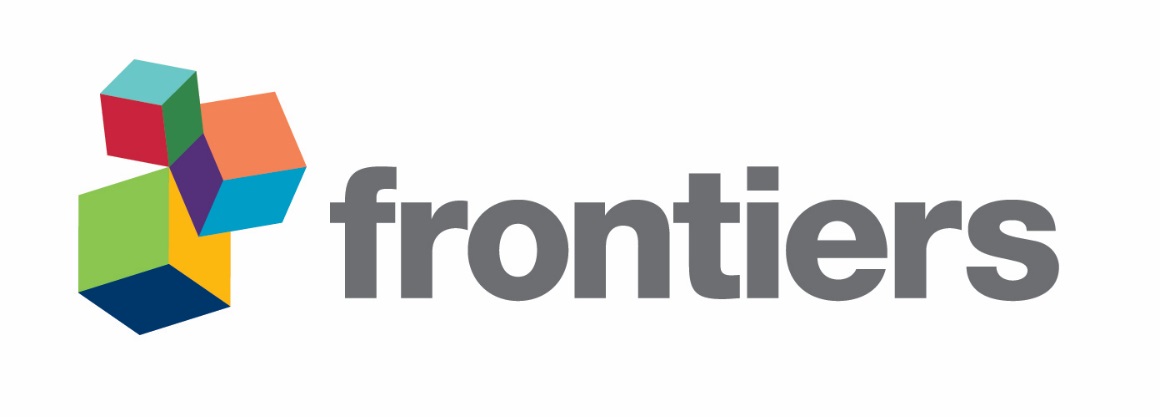
**

**Supplementary Table 1.** Basic information of collected datasets.

**Supplementary Table 2.** Univariable Cox regression analysis of 7 m5C regulatory genes.

**Supplementary Table 3.** GSVA of GO and KEGG between high-risk and low-risk subtypes.

**Supplementary Table 4.** GSEA of GO and KEGG between high-risk and low-risk subtypes.
