# Supplementary material for: Comprehensive Analysis of m5C Methylation Regulatory Genes and Tumor Microenvironment in Prostate Cancer
Source: Front Immunol. 2022 Jun 10;13:914577. doi: 10.3389/fimmu.2022.914577 (PMC9226312; doi:10.3389/fimmu.2022.914577)
Supplement: Supplementary file 2 [file Table_1.docx]

**Supplementary Table S1. Basic information of collected datasets.**

| **Datasets** | **Normal** | **Tumor** |
| --- | --- | --- |
| TCGA | 52 (9.4%) | 499 (90.6%) |
| GSE3325 | 6 (31.6%) | 13 (68.4) |
| GSE55945 | 8 (42.1%) | 11 (57.9%) |
| GSE155056 | 3 (50%) | 3 (50%) |
