# Supplementary material for: Comprehensive Analysis of m5C Methylation Regulatory Genes and Tumor Microenvironment in Prostate Cancer
Source: Front Immunol. 2022 Jun 10;13:914577. doi: 10.3389/fimmu.2022.914577 (PMC9226312; doi:10.3389/fimmu.2022.914577)
Supplement: Supplementary file 3 [file Table_2.docx]

**Supplementary Table S2. Univariable Cox regression analysis of 7 m5C regulatory genes.**

| **Gene** | **HR** | **HR.95L** | **HR.95H** | **pvalue** |
| --- | --- | --- | --- | --- |
| NOP2 | 2.37 | 0.64 | 8.76 | 1.94E-01 |
| NSUN2 | 4.68 | 1.67 | 13.10 | 3.31E-03 |
| TET3 | 3.13 | 1.12 | 8.74 | 2.99E-02 |
| NSUN6 | 1.33 | 0.36 | 4.94 | 6.72E-01 |
| TET1 | 1.88 | 0.56 | 6.34 | 3.11E-01 |
| YBX1 | 3.38 | 1.38 | 8.29 | 7.90E-03 |
| DNMT3B | 2.07 | 0.88 | 4.86 | 9.48E-02 |
