# Supplementary material for: Comprehensive Analysis of m5C Methylation Regulatory Genes and Tumor Microenvironment in Prostate Cancer
Source: Front Immunol. 2022 Jun 10;13:914577. doi: 10.3389/fimmu.2022.914577 (PMC9226312; doi:10.3389/fimmu.2022.914577)
Supplement: Supplementary file 4 [file Table_3.docx]

**Supplementary Table S3. GSVA of GO and KEGG between high-risk and low-risk subtypes.**

| **id** | **logFC** | **adj.P.Val** |
| --- | --- | --- |
| GOBP_REGULATION_OF_PROTEIN_LOCALIZATION_TO_CHROMOSOME_TELOMERIC_REGION | -0.54 | 3.68E-49 |
| GOBP_POSITIVE_REGULATION_OF_TELOMERASE_RNA_LOCALIZATION_TO_CAJAL_BODY | -0.51 | 1.07E-41 |
| GOBP_TELOMERASE_RNA_LOCALIZATION | -0.47 | 1.37E-46 |
| GOBP_IMP_BIOSYNTHETIC_PROCESS | -0.45 | 8.58E-44 |
| GOBP_PROTEIN_LOCALIZATION_TO_NUCLEOPLASM | -0.44 | 3.95E-36 |
| GOBP_REGULATION_OF_ATTACHMENT_OF_SPINDLE_MICROTUBULES_TO_KINETOCHORE | -0.43 | 5.53E-37 |
| GOBP_ESTABLISHMENT_OF_PROTEIN_LOCALIZATION_TO_TELOMERE | -0.43 | 6.70E-47 |
| GOBP_IRES_DEPENDENT_VIRAL_TRANSLATIONAL_INITIATION | -0.42 | 5.90E-37 |
| GOBP_RRNA_CONTAINING_RIBONUCLEOPROTEIN_COMPLEX_EXPORT_FROM_NUCLEUS | -0.42 | 3.30E-50 |
| GOBP_NUCLEOBASE_BIOSYNTHETIC_PROCESS | -0.40 | 3.40E-45 |
| KEGG_AMINOACYL_TRNA_BIOSYNTHESIS | -0.32 | 1.28E-24 |
| KEGG_SPLICEOSOME | -0.28 | 2.08E-29 |
| KEGG_MISMATCH_REPAIR | -0.27 | 6.14E-21 |
| KEGG_GLYOXYLATE_AND_DICARBOXYLATE_METABOLISM | -0.25 | 1.01E-16 |
| KEGG_RNA_DEGRADATION | -0.24 | 1.97E-33 |
| KEGG_BASAL_TRANSCRIPTION_FACTORS | -0.24 | 3.72E-33 |
| KEGG_ONE_CARBON_POOL_BY_FOLATE | -0.24 | 6.72E-25 |
| KEGG_PROTEASOME | -0.23 | 2.11E-10 |
| KEGG_CELL_CYCLE | -0.22 | 2.08E-29 |
| KEGG_CITRATE_CYCLE_TCA_CYCLE | -0.22 | 4.70E-14 |
| KEGG_NUCLEOTIDE_EXCISION_REPAIR | -0.20 | 2.08E-19 |
| KEGG_BASE_EXCISION_REPAIR | -0.20 | 1.01E-13 |
| KEGG_HEMATOPOIETIC_CELL_LINEAGE | 0.20 | 1.23E-12 |
| KEGG_ARACHIDONIC_ACID_METABOLISM | 0.20 | 5.33E-24 |
| KEGG_METABOLISM_OF_XENOBIOTICS_BY_CYTOCHROME_P450 | 0.21 | 8.56E-21 |
| KEGG_GLYCOSPHINGOLIPID_BIOSYNTHESIS_GANGLIO_SERIES | 0.21 | 1.87E-14 |
| KEGG_DRUG_METABOLISM_CYTOCHROME_P450 | 0.22 | 1.72E-24 |
