# Supplementary material for: Comprehensive Analysis of m5C Methylation Regulatory Genes and Tumor Microenvironment in Prostate Cancer
Source: Front Immunol. 2022 Jun 10;13:914577. doi: 10.3389/fimmu.2022.914577 (PMC9226312; doi:10.3389/fimmu.2022.914577)
Supplement: Supplementary file 5 [file Table_4.docx]

**Supplementary Table S4. GSEA of GO and KEGG between high-risk and low-risk subtypes.**

| **Description** | **enrichment Score** | **NES** | **p.adjust** |
| --- | --- | --- | --- |
| GOBP_G_PROTEIN_COUPLED_PURINERGIC_NUCLEOTIDE_RECEPTOR_SIGNALING_PATHWAY | -0.91 | -1.70 | 1.17E-02 |
| GOMF_G_PROTEIN_COUPLED_PURINERGIC_NUCLEOTIDE_RECEPTOR_ACTIVITY | -0.91 | -1.70 | 1.17E-02 |
| GOMF_SIALIC_ACID_BINDING | -0.91 | -1.71 | 1.17E-02 |
| GOCC_T_CELL_RECEPTOR_COMPLEX | -0.89 | -1.72 | 1.15E-02 |
| GOBP_NEGATIVE_REGULATION_OF_INTERLEUKIN_8_PRODUCTION | -0.86 | -1.65 | 1.15E-02 |
| GOBP_POSITIVE_REGULATION_OF_T_CELL_RECEPTOR_SIGNALING_PATHWAY | -0.86 | -1.61 | 1.17E-02 |
| GOBP_POSITIVE_REGULATION_OF_HUMORAL_IMMUNE_RESPONSE | -0.86 | -1.64 | 1.15E-02 |
| GOBP_POSITIVE_REGULATION_OF_CYTOKINE_PRODUCTION_INVOLVED_IN_INFLAMMATORY_RESPONSE | -0.85 | -1.65 | 1.15E-02 |
| GOBP_COMPLEMENT_ACTIVATION_ALTERNATIVE_PATHWAY | -0.85 | -1.59 | 1.17E-02 |
| GOBP_REGULATION_OF_INFLAMMATORY_RESPONSE_TO_ANTIGENIC_STIMULUS | -0.85 | -1.67 | 1.15E-02 |
| GOMF_CCR_CHEMOKINE_RECEPTOR_BINDING | -0.85 | -1.69 | 1.15E-02 |
| GOMF_NUCLEOTIDE_RECEPTOR_ACTIVITY | -0.84 | -1.62 | 1.15E-02 |
| GOBP_T_HELPER_CELL_LINEAGE_COMMITMENT | -0.83 | -1.58 | 1.15E-02 |
| GOBP_POSITIVE_REGULATION_OF_MACROPHAGE_CHEMOTAXIS | -0.83 | -1.58 | 1.15E-02 |
| GOBP_POSITIVE_REGULATION_OF_INTERLEUKIN_2_PRODUCTION | -0.83 | -1.66 | 1.15E-02 |
| GOMF_C_C_CHEMOKINE_BINDING | -0.83 | -1.61 | 1.15E-02 |
| GOBP_CHRONIC_INFLAMMATORY_RESPONSE | -0.83 | -1.60 | 1.15E-02 |
| GOMF_NADPLUS_NUCLEOSIDASE_ACTIVITY | -0.82 | -1.57 | 1.15E-02 |
| GOBP_NEGATIVE_REGULATION_OF_INTERLEUKIN_12_PRODUCTION | -0.82 | -1.57 | 1.15E-02 |
| GOBP_POSITIVE_REGULATION_OF_MONOCYTE_CHEMOTAXIS | -0.81 | -1.57 | 1.15E-02 |
| GOBP_POSITIVE_REGULATION_OF_MACROPHAGE_MIGRATION | -0.81 | -1.61 | 1.15E-02 |
| GOBP_RESPIRATORY_BURST | -0.81 | -1.62 | 1.15E-02 |
| GOMF_G_PROTEIN_COUPLED_CHEMOATTRACTANT_RECEPTOR_ACTIVITY | -0.81 | -1.59 | 1.15E-02 |
| GOBP_NEGATIVE_REGULATION_OF_B_CELL_PROLIFERATION | -0.81 | -1.56 | 1.15E-02 |
| GOBP_POSITIVE_REGULATION_OF_B_CELL_MEDIATED_IMMUNITY | -0.81 | -1.62 | 1.15E-02 |
| GOBP_CD4_POSITIVE_ALPHA_BETA_T_CELL_CYTOKINE_PRODUCTION | -0.81 | -1.55 | 1.15E-02 |
| GOBP_ACTIVATION_OF_PHOSPHOLIPASE_C_ACTIVITY | -0.81 | -1.63 | 1.15E-02 |
| GOBP_DENDRITIC_CELL_CHEMOTAXIS | -0.81 | -1.58 | 1.15E-02 |
| GOMF_IMMUNOGLOBULIN_BINDING | -0.81 | -1.57 | 1.15E-02 |
| GOMF_CHEMOKINE_ACTIVITY | -0.81 | -1.64 | 1.15E-02 |
| GOMF_COMPLEMENT_BINDING | -0.81 | -1.55 | 1.15E-02 |
| GOMF_CHEMOKINE_RECEPTOR_BINDING | -0.81 | -1.64 | 1.15E-02 |
| GOBP_POSITIVE_REGULATION_OF_ANTIGEN_RECEPTOR_MEDIATED_SIGNALING_PATHWAY | -0.80 | -1.56 | 1.15E-02 |
| GOBP_REGULATION_OF_KILLING_OF_CELLS_OF_OTHER_ORGANISM | -0.80 | -1.53 | 1.15E-02 |
| GOBP_PURINERGIC_NUCLEOTIDE_RECEPTOR_SIGNALING_PATHWAY | -0.80 | -1.60 | 1.15E-02 |
| KEGG_B_CELL_RECEPTOR_SIGNALING_PATHWAY | -0.69 | -1.43 | 6.56E-03 |
| KEGG_CALCIUM_SIGNALING_PATHWAY | -0.62 | -1.29 | 6.56E-03 |
| KEGG_CELL_ADHESION_MOLECULES_CAMS | -0.73 | -1.51 | 6.56E-03 |
| KEGG_CHEMOKINE_SIGNALING_PATHWAY | -0.71 | -1.49 | 6.56E-03 |
| KEGG_CYTOKINE_CYTOKINE_RECEPTOR_INTERACTION | -0.73 | -1.54 | 6.56E-03 |
| KEGG_DILATED_CARDIOMYOPATHY | -0.66 | -1.35 | 6.56E-03 |
| KEGG_ECM_RECEPTOR_INTERACTION | -0.67 | -1.38 | 6.56E-03 |
| KEGG_FC_EPSILON_RI_SIGNALING_PATHWAY | -0.64 | -1.33 | 6.56E-03 |
| KEGG_FC_GAMMA_R_MEDIATED_PHAGOCYTOSIS | -0.64 | -1.32 | 6.56E-03 |
| KEGG_FOCAL_ADHESION | -0.62 | -1.31 | 6.56E-03 |
| KEGG_HEMATOPOIETIC_CELL_LINEAGE | -0.75 | -1.55 | 6.56E-03 |
| KEGG_HYPERTROPHIC_CARDIOMYOPATHY_HCM | -0.66 | -1.36 | 6.56E-03 |
| KEGG_JAK_STAT_SIGNALING_PATHWAY | -0.61 | -1.28 | 6.56E-03 |
| KEGG_LEUKOCYTE_TRANSENDOTHELIAL_MIGRATION | -0.65 | -1.36 | 6.56E-03 |
| KEGG_NATURAL_KILLER_CELL_MEDIATED_CYTOTOXICITY | -0.67 | -1.39 | 6.56E-03 |
| KEGG_NEUROACTIVE_LIGAND_RECEPTOR_INTERACTION | -0.64 | -1.34 | 6.56E-03 |
| KEGG_PATHWAYS_IN_CANCER | -0.57 | -1.21 | 6.56E-03 |
| KEGG_REGULATION_OF_ACTIN_CYTOSKELETON | -0.58 | -1.22 | 6.56E-03 |
| KEGG_T_CELL_RECEPTOR_SIGNALING_PATHWAY | -0.69 | -1.44 | 6.56E-03 |
| KEGG_TOLL_LIKE_RECEPTOR_SIGNALING_PATHWAY | -0.66 | -1.36 | 6.56E-03 |
| KEGG_LEISHMANIA_INFECTION | -0.72 | -1.48 | 6.56E-03 |
| KEGG_NOD_LIKE_RECEPTOR_SIGNALING_PATHWAY | -0.75 | -1.53 | 6.56E-03 |
| KEGG_VIRAL_MYOCARDITIS | -0.70 | -1.43 | 6.56E-03 |
| KEGG_COMPLEMENT_AND_COAGULATION_CASCADES | -0.72 | -1.47 | 6.56E-03 |
| KEGG_SYSTEMIC_LUPUS_ERYTHEMATOSUS | -0.79 | -1.59 | 6.56E-03 |
| KEGG_CYTOSOLIC_DNA_SENSING_PATHWAY | -0.73 | -1.48 | 6.56E-03 |
| KEGG_PRIMARY_IMMUNODEFICIENCY | -0.86 | -1.72 | 6.56E-03 |
| KEGG_INTESTINAL_IMMUNE_NETWORK_FOR_IGA_PRODUCTION | -0.80 | -1.60 | 6.56E-03 |
| KEGG_ASTHMA | -0.81 | -1.54 | 1.32E-02 |
| KEGG_ANTIGEN_PROCESSING_AND_PRESENTATION | -0.67 | -1.37 | 2.42E-02 |
| KEGG_ARRHYTHMOGENIC_RIGHT_VENTRICULAR_CARDIOMYOPATHY_ARVC | -0.64 | -1.32 | 2.92E-02 |
| KEGG_TYPE_I_DIABETES_MELLITUS | -0.72 | -1.44 | 3.44E-02 |
| KEGG_ALLOGRAFT_REJECTION | -0.74 | -1.46 | 3.80E-02 |
| KEGG_GRAFT_VERSUS_HOST_DISEASE | -0.74 | -1.46 | 3.80E-02 |
